# Predicting adverse outcomes in adults with a community-acquired lower respiratory tract infection: a protocol for the development and validation of two prediction models for (i) all-cause hospitalisation and mortality and (ii) cardiovascular outcomes

**DOI:** 10.1186/s41512-023-00161-1

**Published:** 2023-12-07

**Authors:** Merijn H. Rijk, Tamara N. Platteel, Geert-Jan Geersing, Monika Hollander, Bert L. G. P. Dalmolen, Paul Little, Frans H. Rutten, Maarten van Smeden, Roderick P. Venekamp

**Affiliations:** 1 Department of General Practice & Nursing Science, Julius Center for Health Sciences and Primary Care, University Medical Center Utrecht, Utrecht University, Utrecht, the Netherlands; 2 Patient and Public Involvement member, Utrecht, the Netherlands; 3 Primary Care Research Center, Primary Care Population Sciences and Medical Education Unit, University of Southampton, Southampton, United Kingdom; 4 Department of Epidemiology & Health Economics, Julius Center for Health Sciences and Primary Care, University Medical Center Utrecht, Utrecht University, Utrecht, the Netherlands

**Keywords:** Lower respiratory tract infection, Cardiovascular disease, Primary care, Electronic Health Record, Prognosis, Prediction model, Hospitalisation, Mortality

## Abstract

**Background:**

Community-acquired lower respiratory tract infections (LRTI) are common in primary care and patients at particular risk of adverse outcomes, e.g., hospitalisation and mortality, are challenging to identify. LRTIs are also linked to an increased incidence of cardiovascular diseases (CVD) following the initial infection, whereas concurrent CVD might negatively impact overall prognosis in LRTI patients. Accurate risk prediction of adverse outcomes in LRTI patients, while considering the interplay with CVD, can aid general practitioners (GP) in the clinical decision-making process, and may allow for early detection of deterioration. This paper therefore presents the design of the development and external validation of two models for predicting individual risk of all-cause hospitalisation or mortality (model 1) and short-term incidence of CVD (model 2) in adults presenting to primary care with LRTI.

**Methods:**

Both models will be developed using linked routine electronic health records (EHR) data from Dutch primary and secondary care, and the mortality registry. Adults aged ≥ 40 years with a GP-diagnosis of LRTI between 2016 and 2019 are eligible for inclusion. Relevant patient demographics, medical history, medication use, presenting signs and symptoms, and vital and laboratory measurements will be considered as candidate predictors. Outcomes of interest include 30-day all-cause hospitalisation or mortality (model 1) and 90-day CVD (model 2). Multivariable elastic net regression techniques will be used for model development. During the modelling process, the incremental predictive value of CVD for hospitalisation or all-cause mortality (model 1) will also be assessed. The models will be validated through internal-external cross-validation and external validation in an equivalent cohort of primary care LRTI patients.

**Discussion:**

Implementation of currently available prediction models for primary care LRTI patients is hampered by limited assessment of model performance. While considering the role of CVD in LRTI prognosis, we aim to develop and externally validate two models that predict clinically relevant outcomes to aid GPs in clinical decision-making. Challenges that we anticipate include the possibility of low event rates and common problems related to the use of EHR data, such as candidate predictor measurement and missingness, how best to retrieve information from free text fields, and potential misclassification of outcome events.

**Supplementary Information:**

The online version contains supplementary material available at 10.1186/s41512-023-00161-1.

## Background

Community-acquired lower respiratory tract infections (LRTI), such as acute bronchitis and pneumonia, are common reasons for primary care consultations. Prognosis is generally favourable, allowing the majority of patients to be managed in primary care with or without antibiotic treatment depending on disease severity and suspected pathogen [1–3]. Adverse outcomes such as hospitalisation or mortality occur in less than 1% of patients with uncomplicated LRTI, i.e. not suggestive of pneumonia, and antibiotics seem not to reduce the occurrence of these outcomes [4]. The risk of complications is far more pronounced in patients with community-acquired pneumonia (CAP) [5], but identifying these patients in primary care can be challenging [6, 7]. In addition, concurrent cardiovascular diseases (CVD) have also been linked to poor prognosis in patients with LRTI, which is supported by recent literature on coronavirus disease 2019 (COVID-19) [8–10].

On the other hand, LRTIs may increase the risk or trigger the occurrence of CVD—and thromboembolic events in particular—such as acute myocardial infarction (AMI), stroke, pulmonary embolism, and deep venous thrombosis for several months after the acute phase of the disease [11, 12]. Activation of the immune system by acute infection is thought to trigger the interaction between inflammatory and prothrombotic pathways (i.e. immunothrombosis) [13]. Infections with respiratory pathogens such as influenza and SARS-CoV-2 have particularly been associated with increased CVD incidence, even in mildly affected patients managed in primary care [14, 15]. For example, the incidence of AMI was found to increase sixfold during the first week after influenza infection [16].

Accurate prediction of the risk of adverse outcomes can aid general practitioners (GP) in identifying LRTI patients in whom close follow-up or (antibiotic) treatment is warranted. Well-known prediction models—such as the Pneumonia Severity Index (PSI) and CURB-65—have been developed in hospitalised patients and primary care validation of these models is hampered by the inclusion of advanced laboratory and radiographic variables [17, 18]. The CRB-65, including confusion, respiratory rate, blood pressure, and age, is proposed as a primary care alternative to predict mortality but has been incompletely validated hampering implementation in primary care [19–22]. Another primary care-derived model including diagnosis, age, heart failure, diabetes, use of oral glucocorticoids, number of hospitalisations in the previous year, and antibiotic use in the previous month to predict hospitalisation and mortality also suffers from limited validation and prediction modelling methods have advanced since the development of both models [8]. A model suitable for routine use in primary care is therefore currently lacking. Here we present the design of the development and external validation of two prediction models aimed at estimating the individual risk of all-cause hospitalisation or mortality (model 1), and short-term incidence of CVD (model 2) in adult primary care LRTI patients using linked routine electronic health records (EHR) data from Dutch primary and secondary care, and the mortality registry.

## Method

### Study design and setting

This prognostic model development and validation study will make use of pseudonymised routine EHR data. The model development cohort will be derived from the Julius General Practitioners’ Network (JGPN) [23], which covers approximately 450,000 Dutch inhabitants, representative of the Dutch population, enlisted in both urban and rural practices in the region of Utrecht. It contains data on patient demographics, consultations (free text including anamnesis, physical examination, and prescribed treatments), coded disease episodes and medical history (using the International Classification of Primary Care (ICPC) [24]), coded prescriptions (using Anatomical Therapeutic Chemical (ATC) codes [25]), data on influenza and pneumococcal vaccinations, coded measurements, and laboratory results. The primary care EHR data will be enriched with linked data on emergency department visits and hospital admissions (from Dutch Hospital Data (DHD) [26]) and mortality (from the National Mortality Registry of Statistics Netherlands (CBS)), resulting in a comprehensive database that covers relevant individual disease trajectories. Details on the various data sources and their coverage are presented in Table 1.

**Table 1:** Details on data sources that will be used for model development and validation

	Characteristics		Type of data					Outcomes			Intended use
Data source	Approximate number of participants	Setting	Demographics	Patient history	Medication use	Measurements (vital and laboratory)	Free text	Hospitalisation	Mortality	Cardiovascular events	D/V
JGPN	0.45M	Primary care	+	+	+	+	+			+	D
DHD	17.5M	Hospital		+				+		+	D and V
National Mortality Registry (CBS)	17.5M	General population	+						+	+	D and V
ANHA	0.6M	Primary care	+	+	+	+	+			+	V

*Abbreviations*: *ICN* Intercity network, *DHD* Dutch Hospital Data, *CBS* Statistics Netherlands, *ANHA* Academic Network of General Practitioners Amsterdam, *M* Million, *D* Development, *V* Validation

### Participants

All patients aged 40 years and older who presented to a GP affiliated to JGPN with an LRTI between 1 January 2016 and 31 December 2019 will be included in the model development cohort. A GP-diagnosed LRTI is defined as the registration of an ICPC code for either pneumonia (R81) or acute bronchitis (R78). Only the first episode of individual patients within the study period will be included. An LRTI-related consultation after a period of 28 days without such consultations is considered a new episode. For external validation, a similarly defined but more recent (i.e. 2022-2023) cohort of primary care LRTI patients will be derived from the Academic Network of General Practitioners from the region of Amsterdam (ANHA) which has a data structure and coverage of healthcare domains similar to JGPN [27].

### Outcomes of interest

**Fig. 1:**
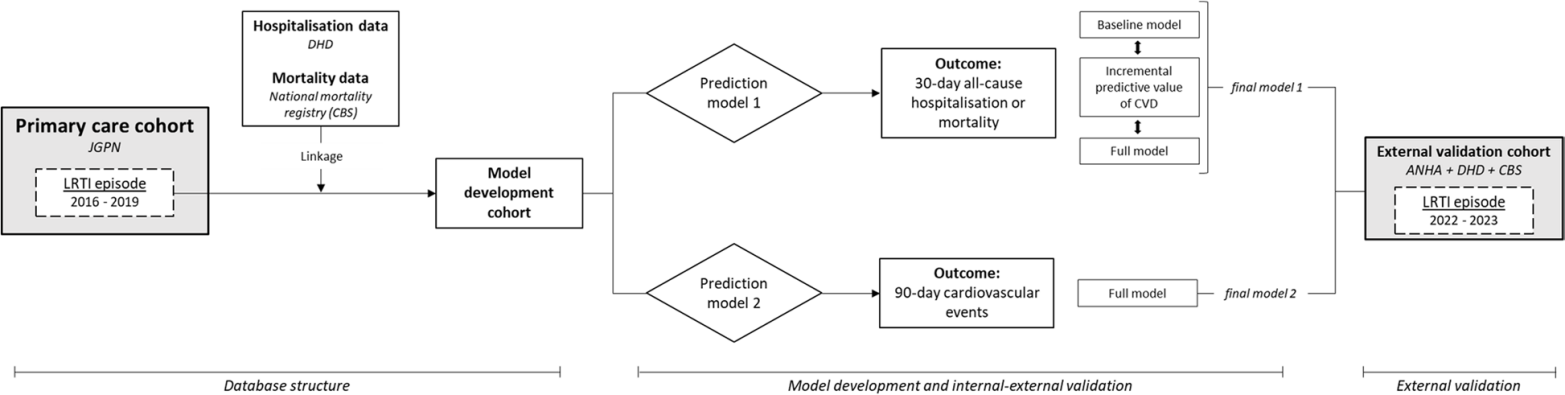
Graphical representation of database structure and anticipated prediction model development process. *Abbreviations*: JGPN, Julius General Practitioners’ Network; LRTI, lower respiratory tract infection; DHD, Dutch Hospital Data; CBS, Statistics Netherlands; ANHA, Academic Network of General Practitioners Amsterdam

We will develop and validate two prediction models that estimate individual risk of 30-day all-cause hospitalisation or mortality (yes/no; model 1) and CVD within 90 days (yes/no; model 2) (Fig. 1).

For model 1, registration of hospitalisation (DHD) and mortality (CBS) within 30 days after LRTI diagnosis will be extracted, irrespective of diagnosis or cause. For model 2, the composite outcome consists of CVD-related mortality and acute (arterial and venous) thromboembolic events within 90 days after LRTI diagnosis: data on CVD-related mortality, acute coronary syndrome, cerebrovascular accident and pulmonary embolism will be extracted from CBS and DHD data using ICD-10 diagnosis codes [28]. Additionally, data on transient ischaemic attack and deep venous thrombosis will be retrieved from DHD (using ICD-10 coding) and JGPN (based on ICPC coding and free text fields of consultations), as these events can be managed in both primary and secondary care. We will additionally explore the option of including exacerbations of heart failure in the outcome for model 2, depending on the feasibility and validity of retrieving such events from primary carefree text data. Since outcome events will be collected from a national registry and follow-up time will be short, the number of missing outcome events will likely be minimal and a non-survival model will be used in our analysis.

### Candidate predictors

Initial candidate predictor selection will be based on a review of the literature and clinical expertise. Candidate predictors will be measured at GP diagnosis of LRTI (i.e. moment of prediction). We will consider the following categories as candidate predictors: demographics (e.g. age, sex), patient history (e.g. smoking status, comorbidities) and chronic medication use (e.g. immunosuppressants, inhalation medication). In addition, we will explore the feasibility of retrieving and—where relevant—the added predictive value of candidate predictor data on signs and symptoms (e.g. shortness of breath, fever), measurements (e.g. oxygen saturation, respiratory rate), and laboratory tests (e.g. point-of-care C-reactive protein (CRP) measured at diagnosis) from free text fields. An overview of all variables that will be considered as candidate predictors can be found in Additional file 1.

### Sample size

Calculations of the required sample sizes for model development are based on estimated event fractions of the outcomes of both prediction models. A total of 15 candidate predictors, interaction terms included, is anticipated. Sample size calculations were performed using the ‘pmsampsize’ package [29] in R version 4.2.2 [30], targeting a maximum shrinkage of 10% to minimize potential overfitting. In the absence of reported R-squared values of previously developed models, we aim at developing models with a minimal anticipated c-statistic (area under the ROC curve) of 0.70. For model 1, the minimal required sample size is 8,635 LRTI episodes assuming a conservative event rate of 3% [1, 22, 31]. For model 2, estimation of the expected cardiovascular event rate is more difficult due to varying definitions and follow-up periods in previous reports [32]. Assuming an event rate of 2.5%, the minimal required sample size is 10,231 LRTI episodes.

The incidence of LRTI in Dutch primary care is estimated at 27.8 episodes per 1000 person-years [33]. With approximately 50% of JGPN participants aged 40 years or older [23] and assuming a similar incidence rate in this population, we anticipate around 25,000 LRTI episodes in our development cohort which would be more than sufficient given our sample size calculations.

### Missing data

Inherent to the use of EHR data, missing data is anticipated. We will consider the absence of registered comorbidities and prescriptions as the absence of the condition or medication use. For all other candidate predictors, we will assess the proportion of cases with missing data and its assumed mechanism. Where appropriate, if the missing at-random assumption is met, missing data will be handled using appropriate techniques, such as multiple imputations with chained equations [34].

### Statistical analysis

Candidate predictor selection will be based on existing literature, clinical expertise, data availability, and a maximum number of candidate predictors according to the sample size calculation. Restricted splines will be considered for continuous predictors, such as age. Both prediction models will be developed using multivariable elastic net regression, accounting for predictor selection during the modelling process.

Model 1 will be developed using an incremental predictive value approach. First, a baseline model (i.e. including age, sex, and an interaction term) will be fitted. Next, the incremental predictive information captured by CVD comorbidity is explored by forcing CVD comorbidities into the model. Finally, a model will be built using all candidate predictors. Model performance of these subsequent models will be compared based on the difference in c-statistics calculated by bootstrapping, change in the distribution of risks, and change in R-squared (pseudo-R-squared), allowing us to choose a final model based on model performance and suitability for use in clinical practice (e.g. a complex model with only a slight increase in performance might not be preferable). For model 2, the final model will directly be developed by forcing all selected candidate predictors into the model. Model performance of the final models will be assessed using quantitative measures of discrimination (c-statistic) and calibration (intercept, slope, and flexible calibration plot), the Cox-Snell R-squared, and decision curve analysis.

The final models will initially be validated through internal-external cross-validation—both geographical and temporal—to assess the heterogeneity of predictor effects by place and time. Subsequently, both final models will be externally validated in the ANHA cohort. Measures of discrimination and calibration will be assessed and, if necessary, the models will be updated using the validation cohort, which would require additional validation [35]. All analyses will be performed in R version 4.2.2 [30], and while reporting we will adhere to the TRIPOD statement (Additional file 2) [36].

## Discussion

In this paper, we present the rationale and design for the development and external validation of two prediction models that can aid GPs in identifying primary care LRTI patients with an increased risk of adverse outcomes. By assessing both the predictive value of CVD for adverse outcomes and the occurrence of cardiovascular outcome events these models specifically address the role of CVD in the prognosis of LRTI.

Currently, available models that stratify LRTI patients based on the risk of poor prognosis are either designed for use in hospitalised patients or suffer from limited validation for use in primary care [8, 19–22, 37, 38], and predicted outcomes are limited to hospitalisation and mortality. In addition, advances in the field of prediction research have led to more sophisticated model development and validation methods. Rather than updating existing models, we therefore aim to include the predictors of these models as candidate predictors while developing new prediction models using state-of-the-art development and validation techniques.

The burden of CVD following an LRTI episode has received considerable attention in hospitalised patients with CAP, and the most frequently observed events include exacerbation of heart failure, atrial fibrillation, and acute coronary syndrome [32, 39]. Some specific respiratory pathogens, such as influenza and SARS-CoV-2, are particularly associated with cardiovascular complications [14, 15, 40]. These observations are not limited to hospitalised patients alone, since several primary care-based studies on patients with RTI also revealed an increased incidence of AMI and stroke up to 90 days after initial infection [41]. Regarding individual prognostic factors, a history of hypertension and a QRISK2 score—a 10-year cardiovascular risk tool—of > 10% were found to be associated with an increased risk of cardiovascular events following an RTI [42]. However, tools for individual risk-stratification of CVD following an LRTI episode in primary care are currently lacking.

Our models will be developed in a period prior to the COVID-19 pandemic. During the first years of the pandemic, aetiology of RTIs was largely reduced to SARS-CoV-2 infection, and circulation of this virus in a naïve population resulted in a wave of severely ill patients [43]. Consequently, hospitalisation and mortality rates among LRTI patients have been relatively high during this period, whereas cardiovascular complications are also more frequent with increasing COVID-19 disease severity [15]. The burden of COVID-19 in the Netherlands has declined ever since as a result of vaccination strategies, natural immunisation after infection and the shift towards the predominance of the less severe Omicron variant. We therefore expect that the epidemiology of primary care LRTIs in the current (post-pandemic) era largely resembles that of the pre-pandemic years in which our models will be developed, which would lead to a stable model performance. This, however, warrants confirmation by our intended external validation in a post-pandemic cohort of primary care LRTI patients.

### Strengths, challenges and limitations

Strengths of the design of our study include the large cohorts used for model development and external validation that derive from a population that is representative of Dutch primary care, the enrichment with linked data on hospitalisation and mortality, and the state-of-the-art assessment of model performance on internal-external and external validation.

Nevertheless, we do anticipate several challenges during the study process. First, the use of routine EHR data requires proper handling of missing data, which in turn depends on the assumed mechanism of missingness [44]. JGPN-affiliated practices receive education on the proper coding of medical history and prescriptions using the ICPC and ATC coding systems, resulting in a rich database. It seems therefore appropriate to consider the absence of such registrations as negative values. Handling of missing data might however be more complex for candidate predictors that will potentially be retrieved from free text fields of index consultations, such as signs, symptoms, measurements, and laboratory test results. We aim to explore the registration of free text-derived parameters and the challenges this introduces in a random sample of the study population prior to embarking on the process of model development with candidate predictors retrieved from free text fields.

A second possible challenge that we consider is a low outcome event rate for model 2 (predicting cardiovascular outcomes), for which we estimated an event rate of 2.5%. If the actual occurrence of CVD following an LRTI episode proves to be lower we may refrain from developing a prediction model, since it is challenging to maintain a high predictive performance in case of rare outcome events [45]. In such an event, we will consider an alternative approach to identify patient and disease characteristics that are associated with an increased risk of CVD following an LRTI by comparing CVD incidence rates among subgroups based on various characteristics, such as patient demographics, comorbidities, and medication use.

Lastly, the use of routine EHR data potentially introduces misclassification on the level of candidate predictors (e.g. medical history), study population (i.e. definition of LRTI episode), and outcome events (e.g. CVD-related mortality). To mitigate potential validity problems due to misclassification, the models should ideally be implemented in a context with similarly structured input data from EHRs. If the models prove to be a safe and valuable addition to the clinical decision-making process, this ultimately results in real-time predicted risks of adverse outcomes in primary care LRTI patients. The potential for developing and implementing such prediction models for LRTI patients is also addressed in the pneumonia guidelines of the American Thoracic Society and Infectious Diseases Society of America [46].

## Conclusion

Community-acquired LRTIs are common in primary care, and patients with increased risk of adverse outcomes are challenging to identify. Currently, existing prediction models for adverse outcomes only focus on hospitalisation and mortality and suffer from incomplete model validation, hampering implementation in clinical practice. The importance of CVD for the prognosis of LRTIs is proposed by both its association with overall poor prognosis and the observed increased incidence of CVD following the initial infection. While considering the interplay between LRTI and CVD, we aim to develop and externally validate two prediction models that predict clinically relevant outcomes such as cardiovascular events, hospitalisation, and mortality. These models can aid GPs in stratifying the risk of poor prognosis in primary care LRTI patients, which may ultimately allow for early detection and prevention of deterioration.

### Supplementary Information

**Additional file 1.** Overview of variables considered as candidate predictors.

**Additional file 2.** TRIPOD Checklist: Prediction Model Development and Validation.

## Data Availability

The data that will be used for this study is available from the Dutch primary care registries (JGPN and ANHA), hospital registry (DHD), and the national mortality registry. Access to these data is restricted and has been granted under license of the current study. Data are therefore only available from the authors upon reasonable request and after formal permission of the respective registries.

## Abbreviations

LRTI: Lower respiratory tract infections
CAP: Community-acquired pneumonia
CVD: Cardiovascular disease
COVID-19: Coronavirus disease 2019
AMI: Acute myocardial infarction
GP: General practitioner
PSI: Pneumonia Severity Index
EHR: Electronic health record
JGPN: Julius General Practitioners’ Network
ICPC: International Classification of Primary Care
ATC: Anatomical Therapeutic Chemical
DHD: Dutch Hospital Data
CBS: Statistics Netherlands (*Dutch: Centraal Bureau voor de Statistiek*)
CRP: C-reactive protein
ANHA: Academic Network of General Practitioners Amsterdam
