# Supplementary material for: Predicting adverse outcomes in adults with a community-acquired lower respiratory tract infection: a protocol for the development and validation of two prediction models for (i) all-cause hospitalisation and mortality and (ii) cardiovascular outcomes
Source: Diagn Progn Res. 2023 Dec 7;7:23. doi: 10.1186/s41512-023-00161-1 (PMC10702048; doi:10.1186/s41512-023-00161-1)
Supplement: Supplementary file 1 — Additional file 1. Overview of variables considered as candidate predictors. [file 41512_2023_161_MOESM1_ESM.pdf]

Additional file 1. Overview of variables considered as candidate predictors

Parameter	*Underlying registrations, if applicable*	*Codes, if applicable*		*Data source*
		ICPC	*ATC*	
**Demographics**				
Age (years)				JGPN
Sex (male, female)				JGPN
Frailty index	Algorithm based on ICPC codes (Drubbel et al., 2013)			JGPN
**Use of medical care**				
Number of GP consultations ≤12 months				JGPN
Hospitalization ≤12 months				DHD
**Vaccination status**				
Pneumococcal conjugate vaccine	Immunisation/preventive medicine of airways	R44	J07BB02 (<5 years)	JGPN
Influenza vaccine	Immunisation/preventive medicine of airways	R44	J07AL01 (<1 year)	JGPN
**Comorbidities: CVD**				
Aortic aneurysm	Aortic aneurysm	K99.01		JGPN
Atrial fibrillation	Atrial fibrillation/flutter	K78		JGPN
Cardiomyopathy	Cardiomyopathy	K84.03		JGPN
Cerebrovascular disease	CVA<br>TIA	K90<br>K89		JGPN
Coronary artery disease	Angina pectoris<br>Other chronic/ischemic heart disease	K74<br>K76		JGPN
Heart failure	Decompensatio cordis	K77		JGPN
Myocardial infarction	Acute myocardial infarction	K75		JGPN
Other arrhythmias	Paroxysmal tachycardia<br>Sick sinus syndrome<br>Wolff-Parkinson-White syndrome<br>Atrioventricular block<br>Long QT-syndrome<br>Presence of pacemaker/ICD	K79<br>K80.03<br>K84.01<br>K84.02<br>K84.07<br>A89.01		JGPN
Pregnancy related CVD	Deep vein thrombosis in pregnancy<br>Postpartum thrombosis	W77.03<br>W99.03		JGPN
Thrombo-embolic disease	Pulmonary embolism<br>Thrombophlebitis/ phlebothrombosis	K93<br>K94		JGPN

Valve disease	Acute rheumatic fever<br>Non-rheumatic valve disease	K71<br>K83		JGPN
**Other comorbidities**				
Smoking status	Algorithm for text mining (Boer et al., 2022)			JGPN
Family history of CVD	Family history of CVD	A29.01		JGPN
Hypertension	High blood pressure<br>Primary hypertension with organ damage<br>Secondary hypertension/organ damage	K85<br>K86<br>K87		JGPN
Atherosclerosis	Atherosclerosis	K91		JGPN
Intermittent claudication	Intermittent claudication	K92.01		JGPN
Rheumatoid arthritis	Rheumatoid arthritis	L88.01		JGPN
History of pneumonia	Pneumonia	R81		JGPN
COPD	Emphysema/COPD<br>Chronic bronchitis/bronchiectasis	R95<br>R91		JGPN
Asthma	Asthma	R96		JGPN
Allergic rhinitis	Allergic rhinitis	R97		JGPN
Adipositas	Adipositas<br>Obesity	T82<br>T83		JGPN
Diabetes mellitus	Diabetes mellitus type 1 and 2	T90		JGPN
Disorders of fat metabolism	Disorders of fat metabolism	T93		JGPN
Renal dysfunction	Renal dysfunction/insufficiency	U99.01		JGPN
Pregnancy related CVD risk factors	Pre-eclampsia<br>Gestational diabetes	W81<br>W84.02		JGPN
Premature menopause	Premature menopause	T99.07		JGPN
Coagulation disorders	Purpura/coagulation disorder/abnormal thrombocytes	B83		JGPN
PCOS	PCOS	T99.06		JGPN
Dementia	Cognitive disorders<br>Dementia	P20<br>P70		JGPN
Thyroid disease	Hyperthyroidism/thyrotoxicosis<br>Hypothyroidism/myxoedema	T85<br>T86		JGPN
IBD	Ulcerative colitis/Crohn’s disease	D94		JGPN
HIV infection	HIV-infection	B90		JGPN
Viral hepatitis	Viral hepatitis	D72		JGPN
Cirrhosis	Cirrhosis/other liver disease	D97		JGPN
Skin ulcer	Ulcer cruris/decubitus/chronic ulcer	S97		JGPN
Gastrointestinal ulcer	Ulcer duodeni	D85		JGPN

	Ulcer pepticum	D86		
Paralysis	Paralysis	N18		JGPN
Hip fracture	Fratcure of collum femoris	L75.01		JGPN
**Oncology**				
Hematologic cancer	Hodgkin lymphoma<br>Non-Hodgkin lymphoma<br>Leukaemia<br>Hematologic/lymphatic malignancy NOS<br>Benign hematologic/lymphatic Neoplasm	B72.01<br>B72.02<br>B73<br>B74<br>B75		JGPN
Digestive tract cancer	Stomach malignancy<br>Colon/rectum malignancy<br>Pancreatic malignancy<br>Digestive tract malignancies NOS	D74<br>D75<br>D76<br>D77		JGPN
Pulmonary cancer	Malignancy of bronchus/lung<br>Other airway malignancy	R84<br>R85		JGPN
Skin cancer	Malignancy skin/subcutis	S77		JGPN
Urological cancer	Malignancy of kidney<br>Malignancy of bladder<br>Other malignancy of urinary tract<br>Malignancy prostate<br>Other urologic malignancy	U75<br>U76<br>U77<br>Y77<br>Y78		JGPN
Gynaecological cancer	Malignancy of cervix uteri<br>Malignancy of breast (female)<br>Other gynaecological malignancies	X75<br>X76<br>X77		JGPN
Other malignancies	Malignancy of unknown primary origin<br>Malignancy of eye/adnexa<br>Malignancy of ear<br>Cardiovascular malignancy<br>Musculoskeletal malignancy<br>Neurologic malignancy<br>Malignancy of thyroid	A79<br>F74.01<br>H75.01<br>K72.01<br>L71.01<br>N74<br>T71		JGPN
**Recent laboratory tests and measurements (timeframe in months prior to LRTI-episode)**				
Body mass index (kg/m^2)	<12 months			JGPN
eGFR (ml/min/1,73 m^2)	<12 months			JGPN
Haemoglobin (mmol/l)	<3 months			JGPN
Leukocytes (x10^9/L)	<6 months			JGPN

BNP or NT-proBNP (pmol/l)	<6 months			JGPN
$HbA1_c$ (mmol/mole)	<6 months			JGPN
TSH (mU/l)	<3 months			JGPN
D-dimer (mg/l)	<3 months			JGPN
LDL (mmol/l)	<6 months			JGPN
**Measurements linked to LRTI-related episode**				
Oxygen saturation (%)				JGPN
Respiratory rate (/min)				JGPN
Heart rate (/min)				JGPN
Systolic blood pressure (mmHg)				JGPN
Diastolic blood pressure (mmHg)				JGPN
Body temperature (°C)				JGPN
C-reactive protein (mg/ml)				JGPN
**Chronic use of medication**[a]				
Recent antibiotic use	Within 1 month prior to LRTI episode		J01	JGPN
Platelet aggregation inhibitors	platelet aggregation inhibitors		B01AC	JGPN
Other anticoagulants	Vitamin K antagonists<br>Dalteparin<br>Enoxaparin<br>Nadroparin<br>Tinzaparin<br>Dabigatran<br>Direct factor Xa inhibitors<br>Fondaparinux		B01AA<br>B01AB04<br>B01AB05<br>B01AB06<br>B01AB10<br>B01AE07<br>B01AF<br>B01AX05	JGPN
Diuretics	Diuretics		C03	JGPN
Beta blockers	Beta blockers		C07A	JGPN
Cardioselective beta blockers	Cardioselective beta blockers		C07AB	JGPN
Calcium channel blockers	Calcium channel blockers		C08	JGPN
ACE-inhibitors	ACE-inhibitors, plain<br>ACE-inhibitors, combinations		C09A<br>C09B	JGPN
ARBs	Angiotensin II receptor blockers, plain<br>Angiotensin II receptor blockers, combinations		C09C<br>C09D	JGPN
Nitrates	Isosorbidemononitrate		C01DA14	JGPN

	Isosorbidedinitrate<br>Nitrogycerin		C01DA08<br>C01DA02	
Lipid-lowering medication	Lipid-lowering agents		C10A	JGPN
Digoxin	Digoxin		C01AA05	JGPN
Flecainide	Flecainide		C01BC04	JGPN
Immunosuppressive drugs	Systemic glucocorticoids<br>Predniso(lo)n ≥7.5mg/day<br>Immunosuppressants		H02AB<br><br>L04A	JGPN
Benzodiazepines	Benzodiazepines		N05CD + N05CF	JGPN
Antidepressants	Antidepressants		N06A	JGPN
Insulin	Insulin and analogs		A10A (-A10AF)	JGPN
Inhalation medication	Long acting beta antagonists (LABA):<br>Formoterol<br>Indacaterol<br>Olodaterol<br>Salmeterol<br><br>Long acting muscarine antagonists (LAMA):<br>Aclidinium<br>Glycopyrronium<br>Tiotropium<br>Umeclidinium<br><br>LABA+LAMA:<br>Aclidinium/formoterol<br>Glycopyrronium/formoterol<br>Indacaterol/glycopyrronium<br>Tiotropium/olodaterol<br>Umeclidinium/vilanterol<br><br>Inhalation corticosteroids (ICS):<br>Beclomethasone<br>Budesonide<br>Fluticasone<br><br>ICS+LABA:<br>Formoterol/beclomethasone<br>Formoterol/budesonide<br>Salmeterol/fluticasone propionate		 R03AC13<br>R03AC18<br>R03AC19<br>R03AC12<br><br><br><br>R03BB05<br>R03BB06<br>R03BB04<br>R03BB07<br><br><br>R03AL05<br>R03AL07<br>R03AL04<br>R03AL06<br>R03AL03<br><br><br>R03BA01<br>R03BA02<br>R03BA05<br><br><br>R03AK08<br>R03AK07<br>R03AK06	JGPN

	Vilanterol/fluticasone furoate<br><br>ICS+LABA+LAMA<br>Beclomethasone/formoterol/glycopyrronium<br>Fluticasone/umeclidinium/vilanterol<br>Formoterol/glycopyrronium/budesonide		R03AK10<br><br><br>R03AL09<br>R03AL08<br>R03AL11	

Abbreviations: CVD, cardiovascular disease; ICPC, International Classification of Primary Care; ATC, anatomical therapeutic chemical; JGPN, Julius General Practitioners' Network; GP, general practitioner; DHD, Dutch Hospital Data; COVID-19, coronavirus disease 2019; CIMS, COVID-vaccination Information and Monitoring System; COPD, chronic obstructive pulmonary disease; PCOS, polycystic ovarium syndrome; NOS, not otherwise specified; IBD, inflammatory bowel disease; HIV, human immunodeficiency virus; LRTI, lower respiratory tract infection; kg, kilogram; m, meter; eGFR, estimated glomerular filtration rate; ml, millilitre; min, minute; mmol, millimole; l, litre; BNP, B-type natriuretic peptide; NT-proBNP, N-terminal (NT)-pro hormone BNP; pmol, picomole; $HbA1_c$, haemoglobin A1c; mU, milliunits; mg, milligram; LDL, low density lipoproteins; mmHg, millimeters of mercury; °C, degrees celcius.

[a] Medication was selected only when commonly used in a primary care setting and is based upon guidelines issued by the Dutch College of General Practitioners and the Dutch pharmacotherapeutic compass.
